# The Mediating Role of Presenteeism in the Relationship Between Psychosocial Safety Climate and Work Engagement

**DOI:** 10.1155/jonm/4081706

**Published:** 2025-12-04

**Authors:** Li Chen, Xiaoli Yang, Yuchen Zhang

**Affiliations:** ^1^ Department of Gastroenterology and Hepatology, West China Hospital, Sichuan University, Chengdu, Sichuan Province, China, scu.edu.cn; ^2^ West China School of Nursing, Sichuan University, Chengdu, Sichuan Province, China, scu.edu.cn; ^3^ Department of Day Surgery, West China Hospital, Sichuan University, Chengdu, Sichuan Province, China, scu.edu.cn; ^4^ Department of Nursing, West China Tianfu Hospital, Sichuan University, Chengdu, Sichuan Province, China, scu.edu.cn

## Abstract

**Background:**

A robust psychosocial safety climate (PSC) can enhance nurses’ work engagement. Conversely, presenteeism—the behaviour of working while ill—reduces nurses’ productivity and negatively impacts their work engagement. However, research is scarce on the interplay between PSC, presenteeism, and work engagement among nurses.

**Objective:**

This study examined the mediation of presenteeism in the relationship between PSC and nurses’ work engagement.

**Methods:**

This cross‐sectional descriptive study was performed among 2982 full‐time registered nurses from tertiary hospitals in 38 cities across 15 provinces in China. An online questionnaire survey was conducted from 2 December 2024 to 31 January 2025. The measurement tools included the 12‐item Psychosocial Safety Climate Scale, the Chinese version of the Stanford Presenteeism Scale, and the Utrecht Work Engagement Scale. The collected data were analyzed using descriptive statistics, independent samples *t*‐tests, one‐way ANOVA, multiple stepwise linear regression analysis, and PROCESS v3.5 macro analysis.

**Results:**

The average scores for PSC, presenteeism, and work engagement were 49.10 (±10.37), 15.23 (±4.67), and 86.28 (±24.49), respectively. Marital status, only‐child status, nurse specialist qualification, PSC score, and presenteeism score significantly influenced nurses’ work engagement, accounting for 43.9% of the variance. PSC boosted work engagement and lowered presenteeism, while presenteeism decreased work engagement. Furthermore, presenteeism partially mediated the relationship between PSC and work engagement.

**Conclusion:**

Nurses report a relatively strong PSC and moderate levels of presenteeism and work engagement. Demographic and work‐related characteristics, along with PSC and presenteeism scores, determine nurses’ work engagement. Furthermore, PSC exerts both a direct and an indirect influence on nurses’ work engagement, with presenteeism serving as a partial mediator.

**Implications for Nursing Management:**

Nurse managers should prioritize fostering a supportive PSC, minimizing presenteeism, and enhancing work engagement. Such measures can ultimately improve care quality and patient safety.

## 1. Introduction

Nurses constitute an essential component of the healthcare workforce. Their physical and mental health, as well as their level of work engagement, directly influence healthcare quality and patient satisfaction. The profession of nursing is characterized by numerous occupational challenges, such as high stress, substantial workload, difficult working conditions, significant occupational risks, frequent night shifts, and low job substitutability. These challenges have a detrimental effect on nurses’ physical and psychological well‐being. In China, the number of registered nurses was approximately 5.84 million by the end of 2024, and this number continues to increase steadily [1]. However, factors such as a rapidly aging population, the introduction of China’s three‐child policy, and the persistent impact of the COVID‐19 pandemic have intensified the demand for nursing services, resulting in a significant shortage of nurses nationwide.

The current state of nursing in China can be effectively analyzed through the lens of the Job Demands‐Resources (JD‐R) model, which categorizes occupational factors as job demands and job resources [2]. According to this model, occupational stress occurs when job demands exceed job resources [3]. In the context of nursing, nurses must exert additional effort when there are limited human and organizational resources coupled with escalating healthcare needs, thus presenting substantial management challenges in clinical practice.

A critical job resource relevant to the nursing profession is the psychosocial safety climate (PSC). It refers to the organization’s commitment to fostering a supportive psychosocial work environment and promoting employees’ psychological health through strategic resource allocation [4]. Previous research has indicated that a robust PSC is an effective organizational intervention, as it enables nurses to cope effectively with increasing workloads [5]. A positive PSC not only fosters constructive workplace behaviors but also mitigates negative behaviors among employees. Furthermore, a strong PSC is associated with improved working conditions [6], increased job satisfaction [7], enhanced employee well‐being [8], and greater work engagement [9–11].

Work engagement refers to a positive psychological state among employees, characterized by high levels of competence, vigor, and professional identification [12]. However, studies have revealed that only approximately 26% of nurses achieve a high degree of work engagement [13]. Freeney and Tiernan [14] conducted a qualitative study among nurses and found that excessive workload is a salient barrier to achieving optimal work engagement. Although Jenaro et al. [15] suggested that departmental allocation and the amount of experience are not significantly associated with nurses’ work engagement, Lepistö et al. [16] reported an incremental increase in work engagement with longer nursing tenure. Furthermore, a longitudinal study revealed higher levels of engagement among nurses when nursing managers adopted less controlling managerial practices [17].

Another critical factor influencing nurses’ performance is presenteeism, defined as the behavior of attending work despite experiencing health problems that justify sick leave [18]. The prevailing shortage of nursing human resources, coupled with continuously rising healthcare demands, places nurses at an elevated risk of presenteeism [19, 20]. Freeling et al. [21] conducted a systematic review to analyze nurse presenteeism in countries such as the United States, Germany, and Sweden. They found prevalence rates ranging from 15.74% to 86.96%. Moreover, a recent global meta‐analysis found an overall prevalence of approximately 49.2% among nursing staff [22]. Furthermore, as nurses served as frontline workers during the COVID‐19 pandemic, they were the most severely impacted by pandemic‐induced anxiety, depression, and distress [23, 24]. Presenteeism adversely affects nurses’ professional competence, concentration, and productivity, potentially increasing the occurrence of nursing errors [25]. Moreover, higher levels of presenteeism significantly reduce the quality of basic nursing care activities, such as nutritional support and rehabilitation interventions [26].

The existing literature has indicated significant associations among PSC, presenteeism, and work engagement. PSC positively predicts work engagement, suggesting that a supportive psychosocial climate acts as a protective mechanism against stress and enhances employee engagement [27–29]. Conversely, PSC is negatively related to presenteeism [5, 30], and presenteeism has been shown to hinder work engagement, with lower engagement levels associated with higher presenteeism [3, 31, 32].

**Figure 1:**
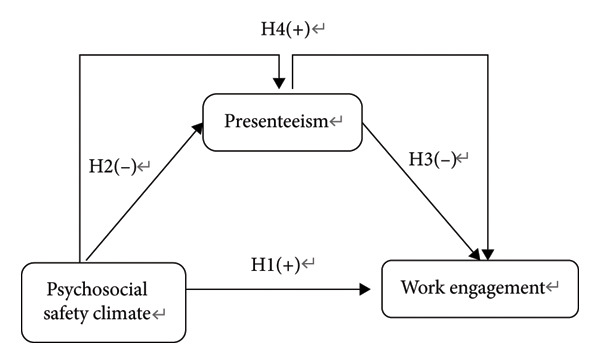
The theoretical framework of the study.

Previous studies have individually explored the relationships between PSC, work engagement, and presenteeism. To our knowledge, no study has collectively examined the relationships among these variables. To address this gap, this study investigates the mediating role of presenteeism in the relationship between PSC and work engagement among Chinese nurses. Figure 1 illustrates the theoretical framework. Guided by the JD‐R model, this study posits the following hypotheses.

 H1: The PSC positively predicts nurses’ work engagement.
 H2: The PSC negatively predicts nurses’ presenteeism.
 H3: Presenteeism negatively predicts nurses’ work engagement.
 H4: Presenteeism mediates the relationship between PSC and nurses’ work engagement.

## 2. Materials and Methods

### 2.1. Design and Participants

This cross‐sectional descriptive study was conducted from 2 December 2024 to 31 January 2025. It targeted full‐time registered nurses working in tertiary hospitals who provided their consent to participate in the study. These individuals were recruited via convenience sampling from tertiary hospitals in 38 cities across 15 provinces in China. We excluded nurses who were enrolled in continuing education programs or internships, as well as those who were on extended leave or vacation during the data collection period. The sample size was determined using the sample estimation method recommended by Kendall [33]. Specifically, we multiplied the number of questionnaire items (72) by 10–20, which resulted in an estimated range of 720–1440 participants. Considering a 20% rate of incomplete or invalid responses, the final sample size was determined to be between 864 and 1728 participants. We distributed 3017 questionnaires.

### 2.2. Data Collection

Data were collected through a questionnaire hosted on “Wenjuanxing” (https://www.wjx.cn), an online survey platform widely used in China. Before initiating data collection, we contacted nursing administrators at the participating hospitals, explained the study’s purpose and procedure, and obtained approval. After obtaining consent, a unique QR code generated by the “Wenjuanxing” platform was sent to the nursing department representatives, who were requested to send the survey invitation to eligible nurses. Nurses who intended to participate were sent the survey link via WeChat or email. The questionnaire outlined the study’s objectives, provided instructions for completion, and assured participants of voluntary participation, anonymity, and confidentiality. Each participant was allowed only one submission and was required to complete the questionnaire within 30 min. Responses submitted in less than 200 s or those containing logical inconsistencies were considered invalid and excluded from analysis.

### 2.3. Measures

#### 2.3.1. Demographic and Work‐Related Characteristics

The questionnaire collected data on participants’ demographic and work‐related characteristics, including sex, age, education level, years of nursing experience, department, marital status, number of children, was the participant an only child, nationality, monthly frequency of night shifts, professional title, job position, engagement in clinical teaching, did the participant possess a nurse specialist qualification, and hospital classification.

#### 2.3.2. PSC

The PSC was assessed using the 12‐item Psychosocial Safety Climate Scale (PSC‐12) developed by Hall et al. [4]. This scale comprises four dimensions (management commitment, management priority, organizational communication, and organizational participation), each consisting of three items. Each item is rated on a five‐point Likert scale (1 = *strongly disagree*, 5 = *strongly agree*), with total scores ranging from 12 to 60. Scores greater than or equal to 42 denote a robust PSC as perceived by employees, while higher scores indicate a stronger PSC. The PSC‐12 has demonstrated good reliability and validity in the Chinese context [34]. In this study, the Cronbach’s *α* coefficient for the PSC‐12 was 0.987.

#### 2.3.3. Presenteeism

Presenteeism was measured using the Chinese version of the Stanford Presenteeism Scale (SPS‐6), adapted by Zhao et al. [35]. This scale evaluates productivity impairment related to health conditions within the past month. It comprises six items across two subscales: work constraints (Items 1–4) and work energy (Items 5 and 6). Each item is rated on a five‐point Likert scale (1 = *strongly disagree*, 5 = *strongly agree*), yielding total scores between 6 and 30. Higher scores represent higher levels of presenteeism and a greater loss of productivity due to health problems. The scale has demonstrated good psychometric properties, with a Cronbach’s *α* coefficient of 0.911, a test–retest reliability of 0.813, and a content validity of 0.835 [35]. In this study, the scale demonstrated a Cronbach’s *α* coefficient of 0.916.

#### 2.3.4. Work Engagement

Work engagement was measured using the original Utrecht Work Engagement Scale (UWES‐17) developed by Schaufeli et al. [36]. The UWES‐17 comprises 17 items distributed across three dimensions: vigor, dedication, and absorption. Each item is rated on a seven‐point Likert scale (1 = *never*, 7 = *always*), yielding total scores that range from 17 to 119. Higher scores indicate greater levels of work engagement. The average item score is used to categorize work engagement as low (average item score < 3), moderate (average item score between 3 and 5), or high (average item score > 5). The Chinese version of the UWES‐17 has been validated by Zhang and Gan [37] and has shown good cross‐cultural applicability, with Cronbach’s *α* coefficients above 0.70. In this study, the Cronbach’s *α* coefficient of the UWES‐17 was 0.977.

### 2.4. Data Analysis

Data were analyzed using IBM SPSS Statistics version 26.0 (IBM Corp., Armonk, NY, USA). Descriptive statistics were presented as means and standard deviations (*M* ± SD) for continuous variables and as frequencies and percentages for categorical variables. Independent‐samples *t*‐tests and one‐way ANOVA were employed to investigate differences in work engagement based on demographic and work‐related characteristics. Variables that showed significant associations in univariate analyses were further analyzed using multiple stepwise linear regression analysis. Furthermore, to analyze the associations between PSC, presenteeism, and work engagement, we tested the mediating effect of presenteeism using Model 4 of the PROCESS v3.5 macro [38]. Bootstrap resampling with 5000 iterations was used to calculate bias‐corrected 95% confidence intervals (CIs). A two‐tailed *p* value of less than 0.05 was considered significant.

### 2.5. Ethical Considerations

This study obtained ethical approval from the Biomedical Ethics Review Committee of West China Hospital, Sichuan University (no. 2024‐1894). Additionally, it strictly adhered to ethical research standards, ensuring participant confidentiality and anonymity. All personally identifiable information was removed from the dataset, and responses were stored securely on password‐protected electronic devices that were accessible only to authorized research personnel. Participation was entirely voluntary, and the participants were informed that they could withdraw from the study at any stage without facing any negative consequences.

## 3. Results

### 3.1. Descriptive Statistics

A total of 3017 questionnaires were collected, of which 2982 were valid, yielding an effective response rate of 98.84%. Among the respondents, 161 (5.4%) were men and 2821 (94.6%) were women. Nurses aged 20–29 years accounted for 36.8% of the participants (*n* = 1098), nurses aged 30–39 years constituted 44.2% (*n* = 1317), and nurses aged 40 years or older represented 19.0% (*n* = 567). Most respondents held a bachelor’s degree (80.0%, *n* = 2387), and 66.8% (*n* = 1992) were married. Regarding professional titles, 39.1% (*n* = 1167) were staff nurses, and 38.5% (*n* = 1149) were charge nurses. Furthermore, approximately half of the respondents identified themselves as clinical teachers. Table 1 presents detailed information on respondents’ demographic and work‐related characteristics.

**Table 1:** Descriptive statistics and univariate analysis of variables related to work engagement (*N* = 2982).

Variables	*n* (%)	*M* ± SD	*t/F*	*p*
Sex			2.416	0.016
Men	161 (5.4)	81.75 ± 24.66		
Women	2821 (94.6)	86.54 ± 24.46		
Age (years)			27.831	< 0.001
20–29	1098 (36.8)	82.06 ± 25.39		
30–39	1317 (44.2)	86.98 ± 24.20		
40–49	452 (15.2)	92.56 ± 22.00		
≥ 50	115 (3.8)	93.94 ± 20.17		
Education level			9.751	< 0.001
Junior college and below	500 (16.8)	81.81 ± 25.04		
Bachelor’s degree	2387 (80.0)	87.16 ± 24.45		
Master’s degree and above	95 (3.2)	87.68 ± 19.54		
Years of nursing experience			23.201	< 0.001
1–5	807 (27.1)	81.42 ± 25.04		
6–10	793 (26.6)	84.31 ± 25.76		
11–15	700 (23.5)	88.38 ± 23.58		
16–20	296 (9.9)	90.74 ± 22.02		
> 20	386 (12.9)	93.28 ± 21.32		
Department			9.464	< 0.001
Internal medicine	824 (27.6)	86.15 ± 25.17		
Surgery	986 (33.1)	86.37 ± 25.14		
Emergency	91 (3.0)	90.43 ± 22.30		
ICU	196 (6.6)	77.56 ± 23.80		
Operating room	348 (11.7)	84.21 ± 22.70		
Other	537 (18.0)	90.14 ± 23.01		
Marital status			28.837	< 0.001
Unmarried	939 (31.5)	81.15 ± 25.62		
Married	1992 (66.8)	88.66 ± 23.53		
Other	51 (1.7)	87.84 ± 26.00		
Number of children			22.277	< 0.001
None	1195 (40.1)	82.01 ± 25.52		
One	1160 (38.9)	90.21 ± 23.38		
Two	598 (20.1)	86.96 ± 23.06		
Three and above	29 (0.9)	91.48 ± 25.8		
Was the participant an only child?			6.160	< 0.001
Yes	1036 (34.7)	90.05 ± 24.44		
No	1946 (65.3)	84.28 ± 24.28		
Nationality			−1.025	0.305
Han nationality	2860 (95.9)	86.19 ± 24.51		
Other	122 (4.1)	88.51 ± 24.05		
Monthly frequency of night shifts			22.309	< 0.001
0	829 (27.8)	91.23 ± 22.40		
1–5	676 (22.7)	86.11 ± 23.52		
6–10	1312 (44.0)	84.29 ± 25.67		
> 10	165 (5.5)	77.93 ± 24.44		
Professional title			20.548	< 0.001
Nurse	388 (13.0)	82.81 ± 25.00		
Staff nurse	1167 (39.1)	84.45 ± 25.63		
Charge nurse	1149 (38.5)	87.37 ± 23.72		
Associate chief nurse and above	278 (9.4)	94.34 ± 19.55		
Engagement in clinical teaching			−0.636	0.525
Yes	1516 (50.8)	86.00 ± 23.98		
No	1466 (49.2)	86.57 ± 25.01		
Did the participant possess a nurse specialist qualification?			5.063	< 0.001
Yes	736 (24.7)	90.02 ± 22.43		
No	2246 (75.3)	85.06 ± 25.01		
Job position				
Clinical nurse	2199 (73.7)	84.70 ± 25.12	10.536	< 0.001
Nursing team leader	193 (6.5)	91.19 ± 23.29		
Head nurse	260 (8.7)	93.25 ± 18.71		
General head nurse	50 (1.7)	91.70 ± 19.90		
Other	280 (9.4)	87.90 ± 24.04		
Hospital classification			3.587	< 0.001
Class III, grade A	2817 (94.5)	86.67 ± 24.34		
Class III, grade B	165 (5.5)	79.65 ± 26.17		

*Note:* *M*, mean; SD, standard deviation; ICU, intensive care unit.

### 3.2. Scores for PSC, Presenteeism, and Work Engagement

PSC, presenteeism, and work engagement showed mean scores (± standard deviation) of 49.10 (±10.37), 15.23 (±4.67), and 86.28 (±24.49), respectively. Table 2 presents the scores for each subscale, along with the corresponding 95% CIs.

**Table 2:** Scores of PSC, presenteeism, and work engagement (*N* = 2982).

Variables	Theoretical score	Total score (*M* ± SD)	Average item score (*M* ± SD)	95% CI
PSC	12–60	49.10 ± 10.37	4.09 ± 0.77	48.68–49.51
Management commitment	3–15	12.26 ± 2.62	4.09 ± 0.76	12.16–12.37
Management priority	3–15	12.38 ± 2.56	4.13 ± 0.69	12.28–12.48
Organisational communication	3–15	12.24 ± 2.54	4.08 ± 0.67	12.13–12.35
Organisational participation	3–15	12.21 ± 2.68	4.07 ± 0.79	12.10–12.32
Presenteeism	6–30	15.23 ± 4.67	2.54 ± 0.78	15.06–15.39
Work constraints	4–20	9.40 ± 5.32	2.35 ± 1.33	9.21–9.59
Work energy	2–10	5.82 ± 2.87	2.91 ± 1.43	5.72–5.93
Work engagement	17–119	86.28 ± 24.49	5.08 ± 1.44	85.40–87.16
Vigor	6–42	29.28 ± 9.28	4.88 ± 1.55	28.94–29.61
Dedication	5–35	26.38 ± 7.19	5.28 ± 1.44	26.12–26.63
Absorption	6–42	30.63 ± 8.62	5.10 ± 1.44	30.32–30.94

*Note:* *M*, mean; SD, standard deviation; PSC, psychosocial safety climate; CI, confidence interval.

### 3.3. Univariate Analysis

Univariate analyses demonstrated significant differences in work engagement based on participants’ demographic and work‐related characteristics. Specifically, work engagement differed significantly based on sex, age, education level, years of nursing experience, department, marital status, number of children, was the participant an only child, monthly frequency of night shifts, professional title, did the participant possess a nurse specialist qualification, job position, and hospital classification (all *p* < 0.05). Table 1 provides the complete results.

### 3.4. Multiple Stepwise Linear Regression Analysis

The multiple stepwise linear regression analysis identified marital status, was the participant an only child, possession of a nurse specialist qualification, PSC score, and presenteeism score as significant predictors of work engagement. Collectively, these variables explained 43.9% of the variance in work engagement scores (see Table 3 for details).

**Table 3:** Multiple stepwise linear regression analysis of variables related to work engagement (*N* = 2982).

Variables	*B*	SE	*β*	*t*	*p*	Tolerance	VIF
Marital status	3.154	1.029	0.061	3.066	0.002	0.481	2.080
Was the participant an only child	−1.648	0.735	−0.032	−2.243	0.025	0.921	1.085
Did the participant possess a nurse specialist qualification	−2.067	0.824	−0.036	−2.508	0.012	0.894	1.119
PSC score	1.313	0.030	0.622	43.344	< 0.001	0.915	1.092
Presenteeism score	−0.271	0.075	−0.052	−3.594	< 0.001	0.911	1.098

*Note:* *R* = 0.665, *R* ^2^ = 0.442, adjusted *R* ^2^ = 0.439; *F* = 156.448, *p < *.001; PSC, psychosocial safety climate.

### 3.5. Mediation Analysis

The mediation analysis confirmed that PSC positively predicted work engagement (*β* = 0.647, *p* < 0.001), thereby supporting Hypothesis 1. PSC negatively predicted presenteeism (*β* = −0.244, *p* < 0.001), thus supporting Hypothesis 2. Furthermore, presenteeism negatively predicted work engagement (*β* = −0.230, *p* < 0.001), thus supporting Hypothesis 3. After presenteeism was introduced as a mediator, PSC continued to predict work engagement significantly. Both the direct and indirect effects (through presenteeism) of PSC were significant (*p* < 0.001), as evidenced by bootstrap 95% CIs that did not contain zero. Specifically, the direct effect (*β* = 0.396) accounted for 96.59% of the total effect (*β* = 0.410), while the mediating effect (*β* = 0.142) of presenteeism contributed 3.41%. Therefore, Hypothesis 4 was also supported. These results are summarized in Tables 4 and 5.

**Table 4:** Mediation analysis between PSC, presenteeism, and work engagement among nurses (*N* = 2982).

Outcome	Variable	*B*	*β*	*t*	*p*	Adjustment *R* ^2^
Work engagement	PSC	4.103	0.647	46.38	< 0.001	0.419
Presenteeism	PSC	−0.089	−0.244	13.73	< 0.001	0.059
Work engagement	Presenteeism	−1.206	−0.230	12.906	< 0.001	0.053

*Note:* PSC, psychosocial safety climate.

**Table 5:** The total, direct, and indirect effects of PSC on work engagement.

Variables	Effect	Boot SE	Boot LLCI	Boot ULCI	Percentage of effect (%)
Total effect	0.410	0.088	3.929	4.276	—
Direct effect	0.396	0.091	3.783	4.138	96.59
Indirect effect	0.142	0.004	0.015	0.031	3.41

*Note:* PSC, psychosocial safety climate; Boot SE: the standard error of the indirect effect estimated by the percentile Bootstrap method with bias correction; Boot LLCI and Boot ULCI refer to the lower and upper bounds of the 95% confidence interval.

## 4. Discussion

This study explored the relationships among PSC, presenteeism, and work engagement among Chinese nurses. The findings indicated a strong PSC, moderate levels of work engagement, and moderate levels of presenteeism in Chinese tertiary hospitals. Multiple factors significantly influenced work engagement, and presenteeism played a partially mediating role in the relationship between PSC and work engagement.

### 4.1. Current Status of PSC, Presenteeism, and Work Engagement

#### 4.1.1. PSC

The mean score for PSC was 49.10 ± 10.37 in this study. The mean scores for items across all four dimensions exceeded four points, indicating that nurses’ overall perception of the PSC and their perceptions across each dimension were at a relatively high level. This mean score was notably higher than the mean score reported by Pien et al. [34] among Taiwanese nurses (34.2 ± 1.9) and the mean score demonstrated by Abdi et al. [39] (30.03 ± 12.1). This difference in the mean scores may be attributed to the fact that the participants in this study worked predominantly in tertiary hospitals, which typically offer better organizational resources and pay greater attention to nurses’ psychosocial needs. It is also associated with hospitals’ increasing emphasis on humanistic care and employee mental health in recent years. Recent studies have shown that interventions like occupational e‐mental health platforms can significantly improve the PSC, reduce occupational stress, and enhance nursing performance [40]. Prioritizing the development of a robust PSC is critical for ensuring healthcare providers’ well‐being and optimal patient outcomes [41]. Thus, nursing managers should consider implementing targeted interventions to foster supportive psychosocial environments.

#### 4.1.2. Presenteeism

The mean score for presenteeism in this study was 15.23 ± 4.67, indicating that nurses’ presenteeism was at a moderate level. Presenteeism constrains nurses’ professional capabilities, preventing them from concentrating fully on their work and thereby reducing efficiency and the quality of care. At present, with the intensification of population aging, the relaxation of the three‐child policy, and the outbreak of the COVID‐19 pandemic in our country, there is a high demand for nurses in society. However, there is a shortage of nursing human resources. Sometimes, nurses had to continue working while ill, resulting in presenteeism. The mean score for presenteeism in this study was similar to the mean score observed in a large‐scale cohort study conducted in various Chinese provinces (15.45 ± 3.87) [42]. However, it was notably lower than the mean score found among nurses in Henan Province (19.49 ± 5.91) [43]. These differences may be due to disparities in hospital characteristics, regional resource availability, and workload intensity. Previous studies have suggested that higher‐tier hospitals often report greater incidences of presenteeism due to increased job demands and pressures [44, 45]. Given the increasing healthcare workforce challenges in populous regions, nursing managers must address presenteeism by enhancing human resource allocation, improving nurses’ health status, and optimizing working conditions.

#### 4.1.3. Work Engagement

The mean total score for work engagement was 86.28 ± 24.49 (average item score = 5.08 ± 1.44), indicating a moderate level of work engagement among nurses. The average item score was higher than that reported in specialized centers in Madinah (4.29 ± 1.04) [46] and in the whole of Saudi Arabia (4.03 ± 1.05) [47]. This difference may be due to the advanced qualifications and clinical capabilities required in Chinese tertiary hospitals. Interestingly, this study showed the highest scores for the “dedication” dimension and the lowest for the “vigor” dimension. These results contradict those of previous studies that reported higher scores for the “vigor” dimension [15, 48]. This difference may have stemmed from cultural factors. Particularly, Confucian culture emphasizes perseverance and commitment, which may have led to higher presenteeism and lower overall vitality due to chronic fatigue and stress [49].

### 4.2. Factors Influencing Nurses’ Work Engagement

Univariate analyses revealed multiple demographic and work‐related characteristics as significant predictors of nurses’ work engagement. Subsequently, a multiple stepwise linear regression analysis was conducted, which identified that marital status, was the participant an only child, did the participant possess a nurse specialist qualification, PSC score, and presenteeism score entered the regression model. Together, these five variables accounted for 43.9% of the variance in work engagement. Our findings align with the results of previous studies that identified various individual, professional, and organizational variables as determinants of work engagement [50, 51]. Given that nurses’ work engagement is influenced by multifaceted and complex factors, tailored management strategies are crucial for effectively addressing individual and organizational needs.

### 4.3. Relationships Among PSC, Presenteeism, and Work Engagement

PSC significantly predicted work engagement (*β* = 0.647, *p* < 0.001). This indicates that higher PSC scores predicted higher levels of work engagement. These findings align with the results reported by Inoue et al. [27]. Dollard et al. [29] found that a strong PSC provides protective and mitigating functions in the development of employee stress, reducing the likelihood of psychological health issues and enhancing employee work engagement. According to the JD‐R model, a favorable PSC enhances nurses’ professional identity. When nurses perceive that nursing managers prioritize their mental health and safety, it stimulates their work enthusiasm, strengthens their sense of meaning and belonging, and consequently elevates their levels of work engagement.

PSC significantly predicted presenteeism (*β* = −0.244, *p* < 0.001). This indicates that higher PSC scores predicted lower frequencies of presenteeism, consistent with findings from previous research [5, 30, 52]. The core causes of presenteeism lie in insufficient human resources, increased demand for nursing services, and high work pressure on nurses. Additionally, the emphasis on dedication in Chinese Confucianism leads nurses to be reluctant to take leave even when they are ill. As a result, although nurses are physically present at work, they are not in a work‐ready state, which leads to frequent occurrences of presenteeism. A favorable PSC can alleviate nurses’ psychological stress, enabling them to confidently request leave when they are ill and in need of rest—thereby reducing the incidence of presenteeism.

Presenteeism significantly predicted work engagement (*β* = −0.230, *p* < 0.001), indicating that more frequent presenteeism among clinical nurses is associated with lower levels of work engagement. Previous research results are consistent with these findings [3, 31, 32]. With the increase in the frequency of presenteeism, the work engagement and efficiency of nurses will decline [53]. According to the recovery theory, presenteeism can lead to the continuous depletion of physical and mental resources, keeping the body in a state of anxiety, fatigue, and tension for a long time [54]. To relieve fatigue, the body needs to mobilize more resources; however, this also leads to continuous consumption of those resources, which can prevent nurses from fully devoting themselves to their work. In this way, their ability to work may decline because they cannot concentrate on completing tasks and may also make oversights and mistakes. Furthermore, if nurses feel overly exhausted, they may develop a negative attitude towards patients, which could affect their level of work engagement.

### 4.4. Mediating Role of Presenteeism

This study uniquely identified presenteeism as a significant mediator in the relationship between PSC and work engagement. Specifically, the mediation analysis confirmed that PSC positively influences work engagement both directly and indirectly (through reduced presenteeism). This suggests that to enhance nurses’ work engagement, attention should be paid not only to the direct impact of PSC but also to the mediating effect of presenteeism. The JD‐R model provides theoretical justification for this mediating effect. At present, there is a shortage of nursing human resources, but the demand for such jobs is high. High job demands cause physical and mental strain on nurses, prompting them to continue working even when they are ill. However, prolonged work while ill may diminish their work enthusiasm and reduce work engagement. Inclusive and harmonious work environments help reduce presenteeism [55, 56]. Moreover, good PSC can enhance both internal and external motivation, improving nurses’ work engagement [57, 58]. Together with the findings of this study, which reveal that presenteeism exerts a partial mediating effect on PSC and work engagement, nursing managers should prioritize initiatives aimed at strengthening the PSC and reducing presenteeism to sustain workforce engagement.

## 5. Strengths and Limitations

This study utilized the theoretical framework of the JD‐R model and a robust mediation analysis approach to elucidate the complex relationships among PSC, presenteeism, and work engagement. Additionally, the large multicenter sampling enhanced the generalizability of the findings within Chinese tertiary hospitals. Nonetheless, this study had several limitations. First, the use of convenience sampling from tertiary hospitals limited the generalizability to nurses in lower‐level healthcare institutions or other contexts. Second, self‐reported measures of PSC, presenteeism, and work engagement may have introduced response bias despite efforts to ensure anonymity. Finally, the cross‐sectional design constrained causal interpretations and may have led to an underestimation of mediation dynamics.

## 6. Recommendations for Future Research

Future studies should employ longitudinal designs to assess the temporal dynamics of the PSC and its sustained impacts on presenteeism and work engagement. Intervention studies exploring digital health platforms that enhance PSC may provide valuable insights into effective strategies for reducing presenteeism. Additionally, observational and qualitative research may offer a deeper understanding of the individual and contextual factors influencing nurses’ work engagement.

## 7. Implications for Policy and Practice

The PSC reflects the organization’s commitment to safeguarding nurses’ psychological health. Nursing managers should prioritize the following.

(a) Developing a supportive organizational climate through interventions that improve mental health and psychological safety.
(b) Maintaining sufficient nursing staff and addressing staffing deficits to alleviate work overload and related presenteeism.
(c) Designing reasonable work schedules and ensuring adequate rest to mitigate the risks of fatigue and burnout.
(d) Encouraging the active involvement of hospital administrators in workforce management.
(e) Promoting self‐care behaviors among nurses through regular health monitoring and healthy lifestyle initiatives.

Implementing these strategies can significantly enhance nurses’ work engagement, optimize patient care quality, and maintain workforce sustainability.

## 8. Conclusions

Nurses in Chinese tertiary hospitals report a strong PSC, moderate levels of work engagement, and moderate levels of presenteeism. Their work engagement is significantly influenced by demographic and work‐related factors, the PSC, and presenteeism. A significant association exists among PSC, presenteeism, and work engagement, with presenteeism partially mediating the relationship between PSC and work engagement. These findings underscore the necessity for nursing managers to strengthen the PSC, proactively manage presenteeism, and implement targeted strategies to enhance nurses’ work engagement and sustain high‐quality patient care.

## Conflicts of Interest

The authors declare no conflicts of interest.

## Funding

This research received no specific grant from any funding agency in the public, commercial, or not‐for‐profit sectors.

## Data Availability

The data that support the findings of this study are available on request from the corresponding author. The data are not publicly available due to privacy or ethical restrictions.
